# Basilar Artery Occlusion Stroke Managed With Tenecteplase Versus Alteplase Before Endovascular Treatment (BAO‐TNK)

**DOI:** 10.1002/acn3.70240

**Published:** 2025-11-14

**Authors:** Rahul R. Karamchandani, Dylan N. Wolman, Tyler M. Bielinski, Nitin Goyal, Jeremy B. Rhoten, Mahesh V. Jayaraman, Christian T. Sidebottom, Bradley A. Gross, Alhamza R. Al‐Bayati, Mohamed F. Doheim, Matthew K. Tobin, Anoop Chinthala, Avi Gajjar, Justin Mascitelli, Matthew R. Webb, Jan‐Karl Burkhardt, Kyle W. Scott, Visish Srinivasan, Daniel Tonetti, Manisha Koneru, David J. Altschul, Dhrumhil Vaishnav, Sumeet Multani, Thana Theofanis, William J. Ares, Je Yeong Sone, Ramin Zand, Sasan Bahrami, Jiang Li, Gary Defilipp, Dale Strong, Radmehr Torabi, Krisztina Moldovan, Kelsey E. Kline, Clemens M. Schirmer, Raul G. Nogueira, Alexandra R. Paul, Bradley N. Bohnstedt, Philipp Hendrix

**Affiliations:** ^1^ Department of Neurology Wake Forest University School of Medicine, Neurosciences Institute, Advocate Health Charlotte North Carolina USA; ^2^ Department of Diagnostic Imaging Warren Alpert School of Medicine at Brown University, Rhode Island Hospital Providence Rhode Island USA; ^3^ Geisinger College of Health Sciences Scranton Pennsylvania USA; ^4^ Department of Neurology, Semmes Murphey Clinic University of Tennessee Health Sciences Center Memphis Tennessee USA; ^5^ Department of Neurology and Neurosurgery University of Pittsburgh School of Medicine Pittsburgh Pennsylvania USA; ^6^ Department of Neurological Surgery Indiana University School of Medicine Indianapolis Indiana USA; ^7^ Department of Neurosurgery Albany Medical Center Albany New York USA; ^8^ Department of Neurosurgery University of Texas Health Science Center at San Antonio San Antonio Texas USA; ^9^ Department of Neurosurgery University of Pennsylvania Health System Philadelphia Pennsylvania USA; ^10^ Department of Neurological Surgery Cooper University Health Care Camden New Jersey USA; ^11^ Department of Neurological Surgery Montefiore Medical Center, Albert Einstein College of Medicine Bronx New York USA; ^12^ Main Line Health‐Jefferson Neurosurgery Paoli Pennsylvania USA; ^13^ Department of Neurosurgery Northshore University HealthSystem Evanston Illinois USA; ^14^ Department of Neurology, College of Medicine The Pennsylvania State University Hershey Pennsylvania USA; ^15^ Department of Molecular and Functional Genomics Weis Center for Research, Geisinger Danville Pennsylvania USA; ^16^ Charlotte Radiology Advocate Health Charlotte North Carolina USA; ^17^ Information and Analytics Services Advocate Health Charlotte North Carolina USA; ^18^ Department of Neurosurgery Warren Alpert School of Medicine at Brown University, Rhode Island Hospital Providence Rhode Island USA; ^19^ Department of Neurosurgery Geisinger Danville Pennsylvania USA

**Keywords:** alteplase, basilar artery occlusion, tenecteplase, thrombolysis

## Abstract

**Objective:**

To compare the effectiveness and safety of tenecteplase (TNK) versus alteplase (TPA) in patients with basilar artery occlusion prior to endovascular treatment (EVT).

**Methods:**

In this retrospective multicenter study (BAO‐TNK), we analyzed consecutive BAO patients from 14 U.S. stroke systems who received TNK or TPA within 4.5 h of last known well and were referred for EVT (01/2020‐08/2024). Multivariable logistic regression models were adjusted for age, sex, NIH Stroke Scale (NIHSS), posterior circulation Alberta Stroke Program Early CT Score (pc‐ASPECTS), last‐known‐well‐to‐door time, and stroke etiology. Outcomes included 90‐day modified Rankin Scale (mRS), reperfusion rates, and intracranial hemorrhage (ICH) per Heidelberg classification.

**Results:**

Of 163 BAO patients, 75 (46.0%) received TNK and 88 (54.0%) received TPA. Rates of 90‐day good functional outcome (mRS 0–3) were comparable between groups (TNK: 61.8% vs. TPA: 48.8%, adjusted odds ratio [aOR] 1.372, 95% CI 0.616–3.054, *p* = 0.439). No significant differences were observed in rates of pre‐thrombectomy early reperfusion (18.7% vs. 14.8%, aOR 0.933, 95% CI 0.369–2.359, *p* = 0.884), post‐thrombectomy final reperfusion (97.3% vs. 92.0%, aOR 2.133, 95% CI 0.376–12.116, *p* = 0.393), 90‐day mortality (32.4% vs. 39.5%, aOR 0.989, 95% CI 0.436–2.244, *p* = 0.979), or symptomatic ICH (4.0% vs. 4.5%, aOR 1.319, 95% CI 0.245–7.114, *p* = 0.747). Predictors of favorable outcome included younger age, lower NIHSS, higher pc‐ASPECTS, shorter LKW‐to‐puncture time, and non‐atherothrombotic stroke etiology.

**Interpretation:**

In BAO stroke, TNK and TPA administered within 4.5 h pre‐EVT were associated with similar functional outcomes, reperfusion success and hemorrhage rates.

## 1 Introduction

Basilar artery occlusion (BAO) stroke comprises about 1% of ischemic strokes and 5%–10% of large vessel occlusions (LVOs), resulting in major disability or death without timely recanalization [1, 2]. For decades, intravenous thrombolysis (IVT) with alteplase (TPA) was the standard of care for eligible patients. However, emerging evidence suggests that moderate to severe BAO stroke patients should be prioritized for EVT, with IVT as adjunctive therapy when eligible, per guideline‐based recommendations [3, 4, 5].

The Tenecteplase versus Alteplase before Endovascular Therapy for Ischemic Stroke (EXTEND IA TNK) trial demonstrated that tenecteplase (TNK), a next generation thrombolytic, achieved significantly higher recanalization rates in anterior circulation LVO stroke compared to TPA, though the study was powered only to test noninferiority rather than superiority, and was notably underpowered for BAO patients with only 6 patients enrolled. While some studies confirmed this finding, others reported comparable reperfusion rates, underscoring the need to further explore confounders such as thrombus properties, onset‐to‐needle and imaging times, collateral status, and occlusion site. In the United States, stroke care has progressively shifted from TPA to TNK, supported by evidence of non‐inferiority, the ease of single‐bolus delivery, and growing clinical experience. This transition was already underway prior to FDA approval, driven by these advantages [6, 7, 8, 9, 10, 11, 12, 13, 14, 15, 16, 17].

The effectiveness of TNK in BAO stroke remains understudied. A 2021 analysis by Alemseged et al. showed higher pre‐thrombectomy recanalization rates with TNK compared to TPA (26% vs. 7%, risk ratio 4.0, 95% CI 1.3–12, *p* = 0.02) with no increase in hemorrhage rates. However, the authors raised limitations, including a small TNK sample size (19/110 patients), heterogeneous TNK dosing regimens (0.25 and 0.4 mg/kg), TNK patients treated predominantly in the randomized controlled trial setting (> 80%) comparing to observational TPA data, and imbalances in drip‐and‐ship transfer rates (TNK 16% and TPA 44%) [18].

Given TNK's established non‐inferiority to TPA in acute ischemic stroke, many U.S. stroke systems increasingly adopted TNK as the primary stroke intravenous thrombolytic in recent years given simplified administration. This transition may be reinforced by the recent FDA approval of TNK for acute ischemic stroke [19]. Despite this shift in practice, the real‐world comparative safety and efficacy of TNK versus TPA in BAO stroke patients undergoing EVT remain unexplored. This study presents a multicenter analysis across 14 U.S. stroke centers, evaluating the functional outcomes of TNK versus TPA in BAO stroke patients referred for EVT.

## 2 Methods

### 2.1 Study Design

The retrospective cohort study was approved by the institutional review board (IRB). All contributing sites had local individual site IRBs in place and data use agreements for de‐identified data were established. Consent was waived due to the retrospective nature of the study. The Strengthening the Reporting of Observational Studies in Epidemiology (STROBE) guideline was followed [20].

The Basilar Artery Occlusion Stroke managed with Tenecteplase versus Alteplase before Endovascular Treatment (BAO‐TNK) study is a retrospective multicenter study with contributions from 14 U.S. stroke systems across nine states. Consecutive BAO stroke patients ≥ 18 years who received IVT and were referred for EVT between 01/2020 and 08/2024 were retrospectively reviewed. Patients were clinically followed until 12/2024. Patients with posterior circulation occlusion stroke not involving the basilar artery were not included.

The management of BAO stroke followed the contemporary American Stroke Association (ASA) guidelines and individual institutional standardized protocols. The indications for TPA (0.9 mg/kg) or TNK (0.25 mg/kg) followed ASA guidelines. The decision to proceed with EVT (arterial catheterization with thrombus aspiration with or without stent‐retriever use), terminate the procedure after first cerebral angiogram showing sufficient reperfusion, or avert the procedure due to rapid neurological improvement was based on the individual clinical team's discretion.

### 2.2 Definition of Study Variables

The primary outcome to assess effectiveness was a good 90‐day functional outcome assessed with the modified Rankin Scale (mRS) score. Good functional outcome was defined as achieving ambulatory function (mRS 0–3) or baseline (pre‐stroke) functional status. Secondary effectiveness outcomes were 90‐day mRS 0–2, early reperfusion and final reperfusion. Early reperfusion was defined as extended thrombolysis in cerebral infarction (eTICI) 2b‐2c‐3 on initial cerebral angiogram, absence of retrievable thrombus, or rapid neurological improvement acutely obviating the need of arterial catheterization. Final reperfusion was defined as EVT resulting in final eTICI 2b‐2c‐3 reperfusion or achieving early reperfusion. The primary safety outcome was all‐cause mortality (90‐day mRS 6). Additionally, secondary safety outcomes included rates of symptomatic intracranial hemorrhage (sICH), parenchymal hematoma and overall intracranial hemorrhage (ICH). Cranial imaging (CT‐head or magnetic resonance imaging) was performed within 24–36 h post‐thrombolysis. Hemorrhage was recorded according to the Heidelberg bleeding classification [21].

The site of occlusion was recorded according to the stroke‐alert CT‐angiography (CTA). All patients had an occlusion of the basilar artery with or without concomitant adjacent vasculature occlusion such as the vertebral or posterior cerebral arteries. Occlusions were classified as vertebrobasilar, basilar, or basilar with posterior cerebral artery (PCA). Collateral scores per BATMAN criteria and posterior circulation Acute Stroke Prognosis Early CT Score (PC‐ASPECTS) were obtained from baseline CTA and CT‐head, respectively [22, 23].

### 2.3 Missing Data and Statistical Analysis

Missing data on one glucose level and 11 blood pressure parameters were imputed using a multiple imputation approach (Multivariate Imputation by Chained Equations package in R). Predictive mean matching was utilized, incorporating selected variables to estimate the missing values. Multivariable binary logistic regression analyses were performed adjusting for age, sex, National Institute of Health Stroke Scale (NIHSS), PC‐ASPECTS, last known well to needle time and stroke etiology. Additionally, best‐fit primary effectiveness (90‐day mRS 0–3) and safety (90‐day mRS 6) outcome models were explored conducting multivariable regression analyses with stepwise backward and forward feature selection. Best‐fit models were chosen based on the lowest Akaike information criterion. In nine cases, the 90‐day mRS endpoint was not available and cases discarded from the corresponding analyses. Analyses were performed using R statistics v4.3 and IBM SPSS v25 with *p* < 0.05 considered significant.

## 3 Results

### 3.1 TNK Versus TPA—Baseline Demographics

A total of 163 patients with BAO were included, of whom 75 (46.0%) received TNK and 88 (54.0%) TPA. Baseline characteristics were broadly comparable between the groups, though female sex was more frequent in the TNK group (53.3% vs. 37.5%, *p* = 0.043), and TNK‐treated patients had lower rates of hypertension (64.0% vs. 79.5%, *p* = 0.027), diabetes (16.0% vs. 34.1%, *p* = 0.008), dyslipidemia (45.3% vs. 61.4%, *p* = 0.041), and smoking history (40.0% vs. 59.1%, *p* = 0.015). Pre‐stroke functional status, baseline stroke severity (NIHSS), site of occlusion, stroke etiology, BATMAN and PC‐ASPECTS, glucose levels, blood pressure ranges, and time since last known well (LKW) to needle and puncture did not differ significantly between groups (Table 1).

**TABLE 1:** Baseline demographics stratified by thrombolytic agent.

Variable	All cases (*n* = 163)	Tenecteplase (*n* = 75)	Alteplase (*n* = 88)	*p*
Age	64 (55–75)	64 (50–72)	66 (57–79)	0.128
Female	73 (44.8%)	40 (53.3%)	33 (37.5%)	0.043
Pre‐morbid mRS ≤ 1	144 (88.3%)	66 (88.0%)	78 (88.6%)	0.900
Hypertension	118 (72.4%)	48 (64.0%)	70 (79.5%)	0.027
Diabetes	42 (25.8%)	12 (16.0%)	30 (34.1%)	0.008
Dyslipidemia	88 (54.0%)	34 (45.3%)	54 (61.4%)	0.041
Atrial fibrilliation	35 (21.5%)	18 (24.0%)	17 (19.3%)	0.468
Cardiac disease	32 (19.6%)	13 (17.3%)	19 (21.6%)	0.495
Previous TIA or stroke	22 (13.5%)	11 (14.7%)	11 (12.5%)	0.687
Ever smoking	82 (50.3%)	30 (40.0%)	52 (59.1%)	0.015
Antithrombotic	60 (36.8%)	24 (32.0%)	36 (40.9%)	0.240
Baseline‐NIHSS (1–42)^b^	20 (9–37)	16 (9–28)	21 (11–26)	0.233
Site of occlusion(s)				
Vertebrobasilar	25 (15.3%)	14 (18.7%)	11 (12.5%)	0.303
Basilar	84 (51.5%)	34 (45.3%)	50 (56.8%)	
Basilar and PCA	54 (33.1%)	27 (36.0%)	27 (30.7%)	
Glucose mg/dl	135 (113–165)	133 (111–165)	137 (117–166)	0.391
Systolic BP (mmHg)	150 (132–171)	150.0 (135–165)	153 (130–179)	0.437
Diastolic BP (mmHg)	84 (73–95)	84 (74–91)	84 (73–95)	0.564
TOAST classification				
Cardioembolic	64 (39.3%)	33 (44.0%)	31 (35.2%)	0.505
Large artery atherosclerosis	34 (20.9%)	15 (20.0%)	19 (21.6%)	
Other	65 (39.9%)	27 (36.0%)	38 (43.2%)	
BATMAN score^a^	7 (5–8)	7 (5–8)	7 (5–8)	0.225
PC‐ASPECTS^a^	10 (9–10)	10 (9–10)	10 (9–10)	0.943
Mothership presentation	74 (45.4%)	39 (52.0%)	35 (39.8%)	0.118
LKW to IVT time [min]	135 (100–193)	129 (98–193)	135 (100–196)	0.790
LKW to puncture time [min]^c^	228 (166–307)	222 (154–320)	252 (175–304)	0.294
Failed VA access [aborted]	3 (1.9%)	1 (1.3%)	2 (2.4%)	0.661

*Note:* Data missing: ^a^(1 TNK), ^b^(5 TPA). Data not applicable: ^c^(4 TNK, 4 TPA).

### 3.2 TNK Versus TPA—Outcomes

The 90‐day functional outcome endpoint was available in 154/163 (94.5%) (Table 2). A good functional outcome (90‐day mRS 0–3) was observed in 42/68 (61.8%) TNK and 42/86 (48.8%) TPA patients (*p* = 0.110). After adjustment, no difference between thrombolytic agents was identified (aOR 1.372, 95% CI 0.616–3.054, *p* = 0.439) (Table 2). Rates of early reperfusion (18.7% TNK versus 14.8% TPA, aOR 0.933, 95% CI 0.369–2.359, *p* = 0.884) and final reperfusion (97.3% TNK versus 92.0% TPA, aOR 2.133, 95% CI 0.376–12.116, *p* = 0.393) were comparable between groups (Table 2). The 90‐day all‐cause mortality was similar between TNK and TPA patients (32.4% vs. 39.5%, aOR 0.989, 95% CI 0.436–2.244, *p* = 0.979). The rates of any ICH (20.9% vs. 21.3%, aOR 1.273, 95% CI 0.559–2.902, *p* = 0.565), parenchymal hematoma (4.9% vs. 4.0%, aOR 1.029, 95% CI 0.190–7.114, *p* = 0.974), and sICH (4.0% vs. 4.5%, aOR 1.319, 95% CI 0.245–7.114, *p* = 0.747) were comparable between TNK and TPA groups, respectively (Table 2).

**TABLE 2:** Multivariable logistic regression analyses of functional, reperfusion, and hemorrhage outcomes.

Variable	All patients (*n* = 163)	Tenecteplase (*n* = 75)	Alteplase (*n* = 88)	Unadjusted *p*	Adjusted OR, 95% CI, *p*
Early reperfusion eTICI 2b‐3	27 (18.4%)	14 (18.7%)	13 (14.8%)	0.505	0.933 (0.369–2.359), *p* = 0.884
Final reperfusion eTICI 2b‐3	154 (94.5)	73 (97.3%)	81 (92.0%)	0.180	2.133 (0.376–12.116), *p* = 0.393
Symptomatic ICH	7 (4.3%)	3 (4.0%)	4 (4.5%)	1.000	1.319 (0.245–7.114), *p* = 0.747
Parenchymal hematoma	8 (4.9%)	3 (4.0%)	5 (5.7%)	0.727	1.029 (0.190–5.570), *p* = 0.974
Any ICH	34 (20.9%)	16 (21.3%)	18 (20.5%)	0.891	1.273 (0.559–2.902), *p* = 0.565
Functional outcome at 90 days^a^					
mRS 0–3	84 (54.5%)	42 (61.8%)	42 (48.8%)	0.110	1.372 (0.616–3.054), *p* = 0.439
mRS 0–2	70 (45.5%)	37 (54.4%)	33 (38.4%)	0.047	1.619 (0.738–3.551), *p* = 0.229
mRS 6	56 (36.4%)	22 (32.4%)	34 (39.5%)	0.401	0.989 (0.436–2.244), *p* = 0.979

*Note:* Adjusted for age, sex, NIHSS, PC‐ASPECTS, stroke etiology and last known well to needle time.

^a^ Data missing in 7 TNK, 2 TPA.

### 3.3 Predictors of Primary Outcome Endpoints

Multivariable regression analysis identified younger age (OR 0.528 per year, *p* = 0.005), lower baseline NIHSS (OR 0.457 per point, *p* < 0.001), higher PC‐ASPECTS (OR 1.693 per point, *p* = 0.014), shorter LKW‐to‐puncture time (OR 0.559 per minute, *p* = 0.012), and non‐atherothrombotic etiology (OR 0.314, *p* = 0.022) to be independently associated with achieving the primary effectiveness endpoint mRS 0–3 at 90 days (Table 3). Regression for the primary safety outcome, 90‐day mRS 6, yielded the same covariables. Mortality was independently associated with older age (OR 1.044 per year, *p* = 0.007), higher NIHSS (OR 1.077 per point, *p* = 0.001), lower PC‐ASPECTS (OR 0.655 per point, *p* = 0.008), longer LKW‐to‐puncture time (OR 1.004 per minute, *p* = 0.013), and atherothrombotic stroke etiology (OR 2.757, *p* = 0.038) (Table 3).

**TABLE 3:** Best‐fit multivariable logistic regression models for primary effectiveness and safety outcomes.

	Odds ratio (95% CI)	*p*
Good functional outcome (mRS 0–3)		
Age (per 1 year)	0.528 (0.332–0.809)	0.005
NIHSS (per 1 point)	0.457 (0.292–0.690)	< 0.001
PC‐ASPECTS (per 1 point)	1.693 (1.126–2.637)	0.014
LKW to puncture (per 1 min)	0.559 (0.336–0.847)	0.012
Atherothrombotic etiology	0.314 (0.112–0.828)	0.022
All‐cause mortality (mRS 6)		
Age (per 1 year)	1.044 (1.013–1.079)	0.007
NIHSS (per 1 point)	1.077 (1.032–1.129)	0.001
PC‐ASPECTS (per 1 point)	0.655 (0.474–0.888)	0.008
LKW to puncture (per 1 min)	1.004 (1.001–1.007)	0.013
Atherothrombotic etiology	2.757 (1.066–7.370)	0.038

## 4 Discussion

This study provides contemporary real‐world evidence from the United States that TNK and TPA yield comparable functional outcomes in BAO stroke patients treated with IVT within 4.5 h of last known well prior to EVT. Additionally, similar rates of early reperfusion, successful final reperfusion, symptomatic and overall ICH rates were observed with TNK compared to TPA. In the Intravenous Tenecteplase Compared With Alteplase for Acute Ischaemic Stroke in Canada (AcT) randomized clinical trial, only 6% (31/520) of LVO strokes were due to BAO [12, 13]. The paucity of BAO data in prior trials renders the findings from this U.S. multicenter cohort valuable in bridging the current evidence gap. Our findings align with and support previous randomized and observational studies that demonstrated the non‐inferiority of TNK compared to TPA in acute ischemic stroke [24]. However, our study does not support the previous preliminary finding that TNK compared to TPA is associated with substantially higher rates of early reperfusion in BAO stroke [18].

At 90 days, ambulatory function was achieved in about 55% of all patients whereas 36% of all patients were deceased, with no significant difference between the TNK and TPA groups. The Endovascular therapy for acute vertebrobasilar occlusion (VERITAS) meta‐analysis pooled individual patient level data from the Basilar Artery International Cooperation Study (BASICS), Basilar Artery Occlusion Endovascular Intervention Versus Standard Medical Treatment (BEST), Endovascular Treatment for Acute Basilar Artery Occlusion (ATTENTION) and Basilar Artery Occlusion Chinese Endovascular Trial (BAOCHE) studies. About 40% of the patients in the thrombectomy‐arm received IVT (TPA or urokinase) and approximately 45% achieved mRS 0–3 and 36% deceased. Importantly, BEST, ATTENTION and BAOCHE were conducted in China and EVT treatment windows were ≤ 8, 12, and 24 h, respectively. Compared to VERITAS, we observed slightly higher rates of mRS 0–3, which may be attributable to shorter LKW‐to‐puncture times and a higher proportion of non‐atherothrombotic stroke etiology, both of which were independent predictors of favorable outcomes alongside younger age, lower NIHSS, and higher PC‐ASPECTS [4, 25, 26, 27, 28].

Additional studies are required to investigate whether extension of the pre‐thrombectomy IVT time windows, additional intra‐arterial thrombolysis, or post‐thrombectomy IVT with TNK compared to TPA could achieve improved reperfusion rates and subsequent superior functional outcomes in BAO stroke. The recently reported ATTENTION‐IA trial did not demonstrate an increase in excellent 90‐day outcomes (mRS 0–1) in posterior circulation thrombectomy patients who received post‐EVT intra‐arterial TNK, though the study was conducted in an exclusively Chinese population with high rates of intracranial atherosclerosis [29]. Extended window IVT with TNK or TPA in patients with a BAO is intriguing, particularly given the results of TRACE III [30] and HOPE [31] suggesting superior functional outcomes without increased death or disability in anterior circulation LVO patients. However, it remains unclear if there is a similar potential adjunctive benefit of late‐window IVT for BAOs given the overall high rates of reperfusion with thrombectomy reported in our study and randomized trials [25, 27]. Finally, whether bridging thrombolysis with TNK yields better outcomes than direct thrombectomy is currently under investigation (BRIDGE‐TNK, NCT04733742; DIRECT‐TNK, NCT05199194). In anterior circulation LVOS, early TNK bridging thrombolysis may improve 90‐day overall disability [32].

Rates of early and final reperfusion may be confounded by clot composition, with BAO clots commonly containing higher red blood cell fractions with relatively fewer platelets compared to anterior circulation LVO [33, 34, 35]. Here, the TNK‐ and TPA‐treated cohorts showed baseline differences in sex, hypertension, diabetes, dyslipidemia and smoking that could have affected clot histology, which in turn can shape the response to thrombolysis, thrombectomy, or their combination. While the presence of these variables did not predict outcome, the combination or a subset of these variables may associate with a clot phenotype disadvantaging early and final reperfusion.

### 4.1 Limitations

Several limitations exist. First, the included data are nonrandomized and the retrospective nature introduces selection bias. Second, treatment decisions remained at the individual stroke teams' discretion and all data were site‐reported. Third, while our sample size represents one of the largest TNK vs. TPA BAO cohorts analyzed to date, it may still be underpowered to detect differences in functional outcomes. Although baseline discrepancies between groups were acknowledged, the reasons why functional outcomes tended to numerically favor the TNK group remain unclear. The potential impact of differences in clot composition between cohorts, and consequently on reperfusion success, could not be evaluated. Fourth, early reperfusion with rapid improvement after IVT may have been missed due to the absence of prospective data collection. Finally, despite restricting data inclusion to the most recent 5 years, potential temporal bias cannot be excluded. We did not observe a time‐trend association with assessed outcomes, although the evolving EVT utilization over this period may have influenced decision‐making about EVT candidacy.

## 5 Conclusion

This U.S. multicenter study provides real‐world evidence that TNK and TPA achieve comparable functional outcomes in BAO patients treated with IVT within 4.5 h of time LKW and referred for EVT. Additionally, similar rates of early reperfusion, successful final reperfusion, and symptomatic and overall intracranial hemorrhage rates were observed with TNK compared to TPA.

## Author Contributions

P.H. contributed to the conception and design of the study; R.R.K., D.N.W., T.M.B., N.G., J.B.R., M.V.J., C.T.S., B.A.G., A.R.A.‐B., M.F.D., M.K.T., A.C., A.G., J.M., M.R.W., J.‐K.B., K.W.S., V.S., D.T., M.K., D.J.A., D.V., S.M., T.T., W.J.A., J.Y.S., R.Z., S.B., J.L., G.D., D.S., R.T., K.M., K.E.K., C.M.S., R.G.N., A.R.P., B.B., and P.H. contributed to the acquisition and analysis of data; R.R.K, D.N.W, J.L. and P.H. contributed to drafting the text or preparing the figures.

## Conflicts of Interest

Outside the work presented here, R.R.K. received research support from Genentech and R.G.N. consulting fees for an advisory role with Genentech. The other authors declare no conflicts of interest.

## Data Availability

Deidentified, aggregate data will be made available upon reasonable request to qualified investigators with approval by the authors. Requests can be made to the corresponding author by email.
